# Tumor pH-responsive metastable-phase manganese sulfide nanotheranostics for traceable hydrogen sulfide gas therapy primed chemodynamic therapy

**DOI:** 10.7150/thno.42981

**Published:** 2020-01-20

**Authors:** Ting He, Xialing Qin, Chao Jiang, Dawei Jiang, Shan Lei, Jing Lin, Wei-Guo Zhu, Junle Qu, Peng Huang

**Affiliations:** 1 Marshall Laboratory of Biomedical Engineering, International Cancer Center, Laboratory of Evolutionary Theranostics (LET), School of Biomedical Engineering, Shenzhen University Health Science Center, Shenzhen 518060, China.; 2 Key Laboratory of Optoelectronic Devices and Systems of Ministry of Education and Guangdong Province, College of Optoelectronic Engineering, Shenzhen University, Shenzhen 518060, China.; 3 Guangdong Key Laboratory of Genome Instability and Human Disease Prevention, Carson International Cancer Center, Department of Biochemistry and Molecular Biology, Shenzhen.; University School of Medicine, Shenzhen 518055, China.

**Keywords:** manganese sulfide, hydrogen sulfide, gas therapy, chemodynamic therapy, nanotheranostics.

## Abstract

Manganese-based nanomaterials have piqued great interest in cancer nanotheranostics, owing to their excellent physicochemical properties. Here we report a facile wet-chemical synthesis of size-controllable, biodegradable, and metastable γ-phase manganese sulfide nanotheranostics, which is employed for tumor pH-responsive traceable gas therapy primed chemodynamic therapy (CDT), using bovine serum albumin (BSA) as a biological template (The final product was denoted as MnS@BSA). The as-prepared MnS@BSA can be degraded in response to the mildly acidic tumor microenvironment, releasing hydrogen sulfide (H_2_S) for gas therapy and manganese ions for magnetic resonance imaging (MRI) and CDT. *In vitro* experiments validated the pH-responsiveness of MnS@BSA at pH 6.8 and both H_2_S gas and •OH radicals were detected during its degradation. *In vivo* experiments showed efficiently tumor turn-on *T*_1_-weighted MRI, significantly suppressed tumor growth and greatly prolonged survival of tumor-bearing mice following intravenous administration of MnS@BSA. Our findings indicated that MnS@BSA nanotheranostics hold great potential for traceable H_2_S gas therapy primed CDT of cancer.

## Introduction

Cancer nanotheranostics provide a new solution for cancer management using nanotechnology to integrate medical imaging and therapy of cancer 1-7. Magnetic resonance imaging (MRI) is a classical noninvasive medical imaging that benefits high spatial resolution without ionizing radiation and tissue depth limits, which is widely employed in MRI-based nanotheranostics 8-12. For example, Gadolinium (Gd)-nanomaterials and iron oxide nanoparticles have been developed for longitudinal (*T*_1_) and transverse (*T*_2_) MR contrast agents, respectively 13-18. Recently, manganese-based MR contrast agents, such as manganese sulfide (MnS) 19-21, manganese oxide 22, manganese dioxide 23-27, manganese carbonate 28, and manganese iron 29-30, have been widely explored in the field of cancer theranostics. The MnS are mainly included three crystal formations: α-phase, β-phase and γ-phase. The α-phase MnS is highly stable and non-degradable, raising the potential long-term toxicity that hampers its further biomedical application 31-32. On the contrary, γ-MnS is metastable and can be degraded in the acidic microenvironment and release manganese ions (Mn^2+^). Therefore, the development of γ-MnS-based nanotheranostics is promising for tumor pH-responsive* T*_1_-weighted MRI of cancer.

For cancer therapy, chemodynamic therapy (CDT) can convert hydrogen peroxide (H_2_O_2_) into the toxic hydroxyl radical (•OH) at tumor tissues, thus killing tumor cells 33-35. Recently, it has been reported MnO_2_ shell was decomposed in tumor microenvironment to release Mn^2+^, which can catalyze H_2_O_2_ to produce •OH at the presence of HCO_3_^-^ in physiological environments by a Fenton-like reaction manner as that catalyzed by iron ions 36. This process consumes glutathione (GSH) and enhances CDT effect simultaneously. Therefore, we proposed the released Mn^2+^ from tumor pH-responsive γ-MnS nanoparticles could be also used for CDT of cancer.

Additionally, gas therapy is an emerging therapeutic strategy based on the bioeffects of several kinds of gases, such as nitric oxide (NO), carbon monoxide (CO), hydrogen sulfide (H_2_S) and hydrogen (H_2_) 37-39. H_2_S gas is an important endogenous bio-signaling molecule, similar to NO and CO 38, 40-42,and exhibits concentration-dependent biological effects 43-44. It can be metabolized in mitochondria at nanomolar (nM) concentrations, while at micromolar (µM) concentrations, H_2_S gas shows a substantial anti-proliferative effect on MCF-7 breast cancer cells 45. The anticancer effect of H_2_S gas has also been reported in living organisms 45-46. However, traditional H_2_S donors, such as Na_2_S and NaHS, the H_2_S gas release is too fast to maintain a long-term effect, while organic compounds, such as GYY4137, are too complicated to obtain 46. Therefore, the development of novel H_2_S donors is highly desirable for cancer gas therapy.

**Scheme 1:**
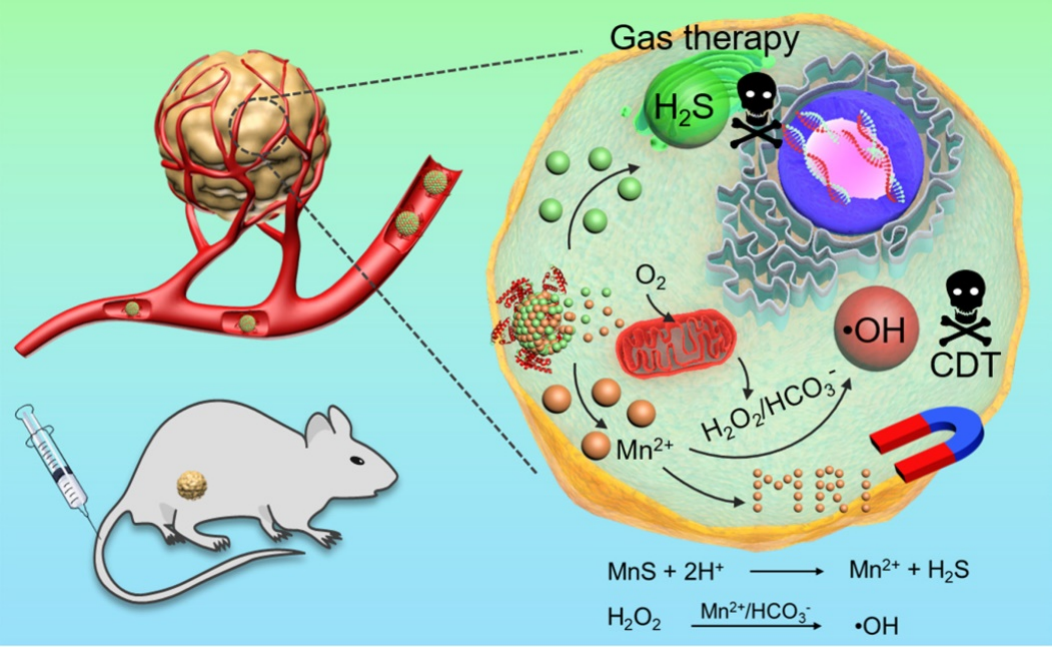
Metastable-phase manganese sulfide nanotheranostics (MnS@BSA) for tumor pH-responsive traceable hydrogen sulfide (H_2_S) gas therapy primed chemodynamic therapy (CDT) of cancer. The MnS@BSA can be degraded in response to the mildly acidic tumor microenvironment, releasing H_2_S for gas therapy and Mn^2+^ for magnetic resonance imaging (MRI) and CDT of cancer.

The combination of CDT and H_2_S gas therapy into a single nanoplatform can achieve enhanced anticancer effect. Because H_2_S gas is a signal molecule that, like NO gas, can cause vasodilation and reduce vascular tension in solid tumors. Herein, we developed a facile wet-chemical method to synthesize size-controllable, biodegradable, and metastable γ-phase MnS using bovine serum albumin (BSA) as a template for tumor pH-responsive* T*_1_-weighted MRI guided the integration of CDT and gas therapy (**Scheme 1**). BSA is used to regulate the size of MnS nanoparticles by tuning the ratio of BSA and Mn^2+^. The as-prepared MnS@BSA is tumor pH-responsive and will be dissociated in acidic tumor microenvironment, producing H_2_S gas and releasing Mn^2+^ ions simultaneously. The former can be used for gas therapy while the latter can provide CDT and serve as a contrast agent for *T*_1_-weighted MRI.

## Experimental Section

### Synthesis of MnS@BSA

MnS@BSA were synthesized by gradually adding Mn(NO_3_)_2_ to a Na_2_S solution. Typically, different amounts of BSA (2.5, 5, and 10 mg) was added into 400 µL of Na_2_S (0.5 M). The volume was later set to 40 mL using deionized water and pH was adjusted to 7.4-7.8 using 0.5 M H_2_SO_4_. After 5 min of vacuuming to remove O_2_, the system was protected by N_2_. Then 2 mL of Mn(NO_3_)_2_ (0.05 M) was added to the mixed solution by a micro-injection pump in 30 min. A flesh pink product was produced, washed and concentrated by centrifugation (12000 rpm, 30 min). The obtained nanoparticles were named as MnS@BSA-2.5, MnS@BSA-5 and MnS@BSA-10. The MnS@BSA-10 with uniform size was used for further studies.

### Levels of Mn^2+^ and H_2_S Released from MnS@BSA

For Mn^2+^, 400 μL of MnS@BSA (5.5 mM) was added into a 3500 Da dialysis bag with PBS buffer (50 mL 10 mM) at pH 6.8 and 7.4, respectively. The solutions were kept stirring at 37 °C and 2 mL was removed for analysis at 0, 0.5, 1, 2, 4, 8, 12, 24, 48, and 72 h. Mn element quantification was performed by using an ICP-AES. For H_2_S, 400 μL of MnS@BSA (5.5 mM) was added into a 3500 Da dialysis bag with HEPES buffer (50 mL 10 mM) at pH 6.8 and 7.4, respectively. 1 mL of the solution was removed as a working solution for H_2_S analysis at 0, 1, 2, 5, 10, 20, 30, 60 min. H_2_S concentration was analyzed using a standard MB method^43^. Briefly, 100 μL of the working solution, 100 μL of 1% (w/v) Zn(OAc)_2_, 20 μL of DMPD (20 mM) and 20 μL of FeCl_3_ (30 mM) were mixed, kept at room temperature for 15-20 minutes, and the optical density (OD) at 663 nm was measured.

### Hydroxyl Radical Catalyzed by MnS@BSA

MnS@BSA or MnCl_2_ was added into 10 μg/mL MB solution which containing 25 mM NaHCO_3_ and 10 mM H_2_O_2_. The OD@665 nm of the above mixture solution was monitored by UV-Vis spectrophotometer at different time points from 0 to 60 min. For ESR detection, 25 mM of NaHCO_3_ were used as the solvent for H_2_O_2_ (10 mM) and DMPO (100 μM). Five groups were tested: H_2_O_2_, MnS@BSA, MnCl_2_, H_2_O_2_ + MnS@BSA, and H_2_O_2_ + MnCl_2_. The concentrations of MnCl_2_ and MnS@BSAwere set to 5 μM and the total volume was 0.5 mL. All reagents were mixed and kept for 30 min before hydroxyl radicals were measured.

### *In vitro* Combination Therapy

MCF10A and 4T1 cells were seeded into 96-well plates at a density of 5000 cells per well. After 12 h in the incubator, the cells were incubated with MnS@BSA, MnCl_2_, Na_2_S ([Mn]/[S] = 0-200 μM) for 24 h. To assess the cytotoxicity of MnS@BSA, various concentrations of antioxidant lascorbic acid (AA, 0-80 μM) in 10 µM with different concentration of MnS@BSA ([Mn]=0-200 μM) were tested. The standard MTT assay was carried out to evaluate the cell viability.

### Hydroxyl Radicals and H_2_S in Cells

5×10^5^ of 4T1 cancer cells were digested and resuspended into 2 mL of DMEM and subcultured into φ15 confocal laser scanning microscopy (CLSM)-exclusive culture disks for another 12 h. Subsequently, the medium was removed and the disks were rinsed by PBS twice before adding 1 mL of DMEM containing 50- 200 μM of MnS@BSA, 200 μM of MnCl_2_. Finally, the medium was removed and the fluorescence probe addition: 25 μM of non-fluorescent DCFH-DA was added to reduce hydroxyl radical for 30 min, then the fluorescence of DCF was observed on CLSM. For H_2_S, 2.5×10^5^ 4T1 cancer cells were subcultured into 24-well plates for 12 h. After incubated with 100 μM of the WPS-5 probe for 30 min, then treated with 0, 50, 100, 200 μM of MnS@BSA and 200 μM of Na_2_S. Then added HEPES containing 100 μM of CTAB (pH 7.4,). After 10 min, fluorescence images were acquired.

### *In vivo* Imaging and Biodistribution

Tumor-bearing mice were scanned with a 3T clinical United Imaging 790 MRI scanner (United Imaging, Shanghai, China) before and after intravenous administration of 2.5 mg/kg of Mn (MnS@BSA or MnCl_2_). T_1_-weighted images were acquired by FSE sequence at 0, 1,2, 4, 8, and 24 h and the following parameters were applied: TR=700 ms, TE=14.3 ms, Flip Angle=145 º, matrix size, 160 x 160, slice thickness, 1.5 mm. Signal intensities were measured in defined regions of interest (ROIs) with software named Image J. MRI were performed at 0, 1, 2, 4, 8, 24 h post-injection.

Six tumor-bearing mice were divided into two groups (n=3). After administration of 2.5 mg/kg of MnS@BSA through their tail veins. One group was euthanized after 4 hours, and the other was euthanized after 24 hours. Tumors and major organs (heart, liver, spleen, lung, kidney and muscle) were obtained and washed with PBS. Each organ was immersed in 3 mL of nitric acid overnight, and then heated to 150 ℃. The final volume was set to 1.5 mL. The concentration of Mn was measured by ICP-AES to estimate the *in vivo* bio-distribution of MnS@BSA.

### *In vivo* Combination Therapy

The therapeutic effects of MnS@BSA were examined on 4T1-Luciferase mammary tumor xenograft on BALB/c mice. Tumor-bearing mice were randomly divided into seven groups: 5 mice per group, administered dose was 2.5 mg/kg MnS@BSA. The seven groups were: PBS, MnCl_2_, Na_2_S, MnS@BSA -2.5, MnS@BSA -5, MnS@BSA -10 and MnS@BSA -10+AA. Their body weights and tumor volumes were measured every two days to evaluate the therapeutic performance. FL imaging of mice were recorded on day 2, 4, 6, 8 and 14 post-treatments. Before FL imaging, 15 mg/mL of D-luciferase potassium salt was intraperitoneally injected at a dose of 10 μL/g, and bioluminescence imaging was performed at 10-15 min post-injection for tumor growth evaluation.

## Results and Discussions

### Preparation and Characterization of MnS@BSA

**Figure 1:**
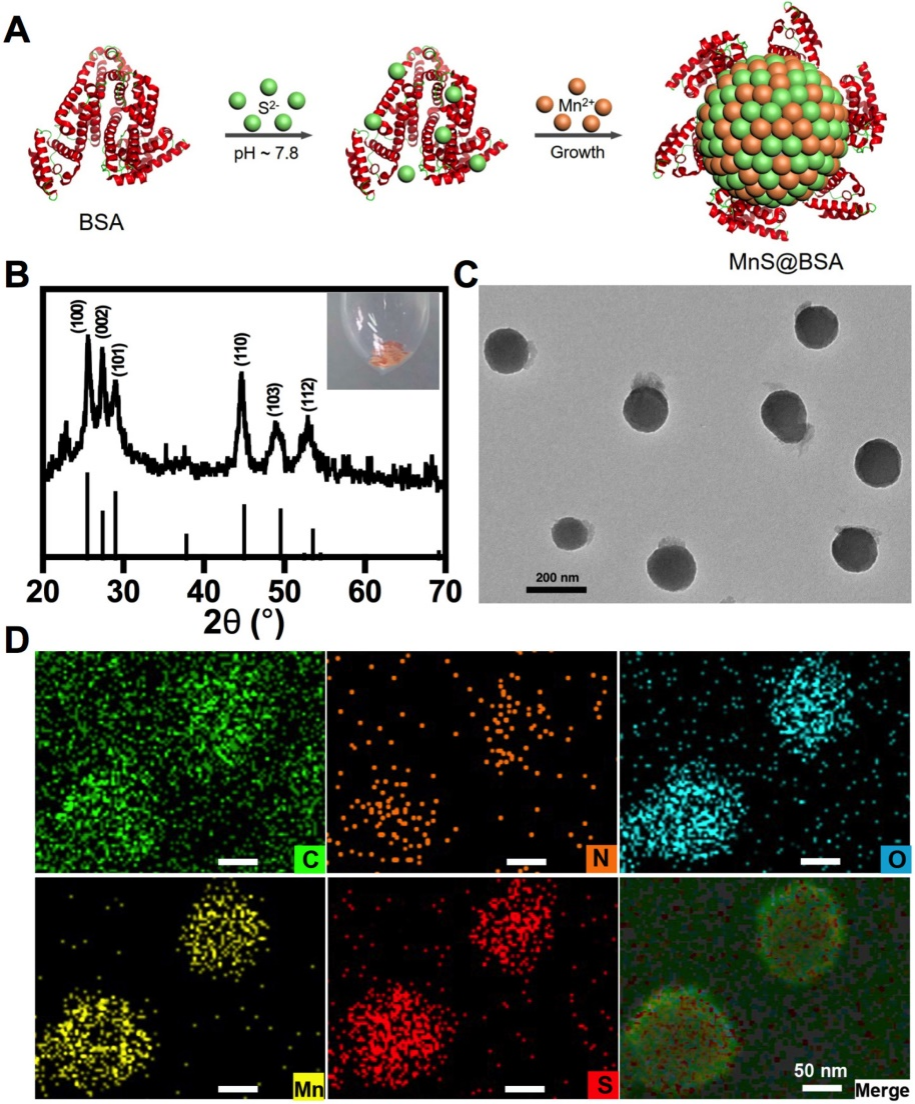
(A) The schematic illustration of the synthesis of metastable phase MnS@BSA. MnS@BSA (B) XRD pattern of MnS@BSA, (C) TEM image of MnS@BSA, scale bar 200 nm, (D) Element mapping images of MnS@BSA. Scale bar 50 nm.

The α-MnS was commonly synthesized by the high temperature decomposition of manganese oleate in oil phase 19, 47-48. It was not water-soluble and needed further surface modification for biomedical applications. In our case, MnS@BSA was synthesized by a wet-chemical method (**Figure 1A**). Briefly, manganese source (MnCl_2_) was slowly added to a mixture solution of Na_2_S and BSA, which was vacuumed and protected with N_2_ gas to obtain a pink solution (**Figure S1**), the typical color of γ-phase MnS. XRD pattern (JCPDS Card No.40-1289) further confirmed that MnS@BSA is γ-phase MnS (**Figure 1B**). As shown in **Figure 1C,** MnS@BSA exhibited well-defined sphere. The size of MnS@BSA can be controlled from 300 to 150 nm by adjusting the amount of BSA from 2.5 to 10 mg. More BSA were added, the size of MnS@BSA were smaller (**Figure S2**). The MnS@BSA (10 mg of BSA) with the smallest size (~ 150 nm) was selected for the following experiments. As shown in the element mapping of MnS@BSA (**Figure 1D**), C, N and O elements were assigned to BSA, S and Mn elements were assigned to MnS. These results indicated that γ-phase MnS was successfully synthesized using BSA as template, which can improve its solubility and biocompatibility.

### Hydroxyl Radicals and H_2_S of MnS@BSA

**Figure 2:**
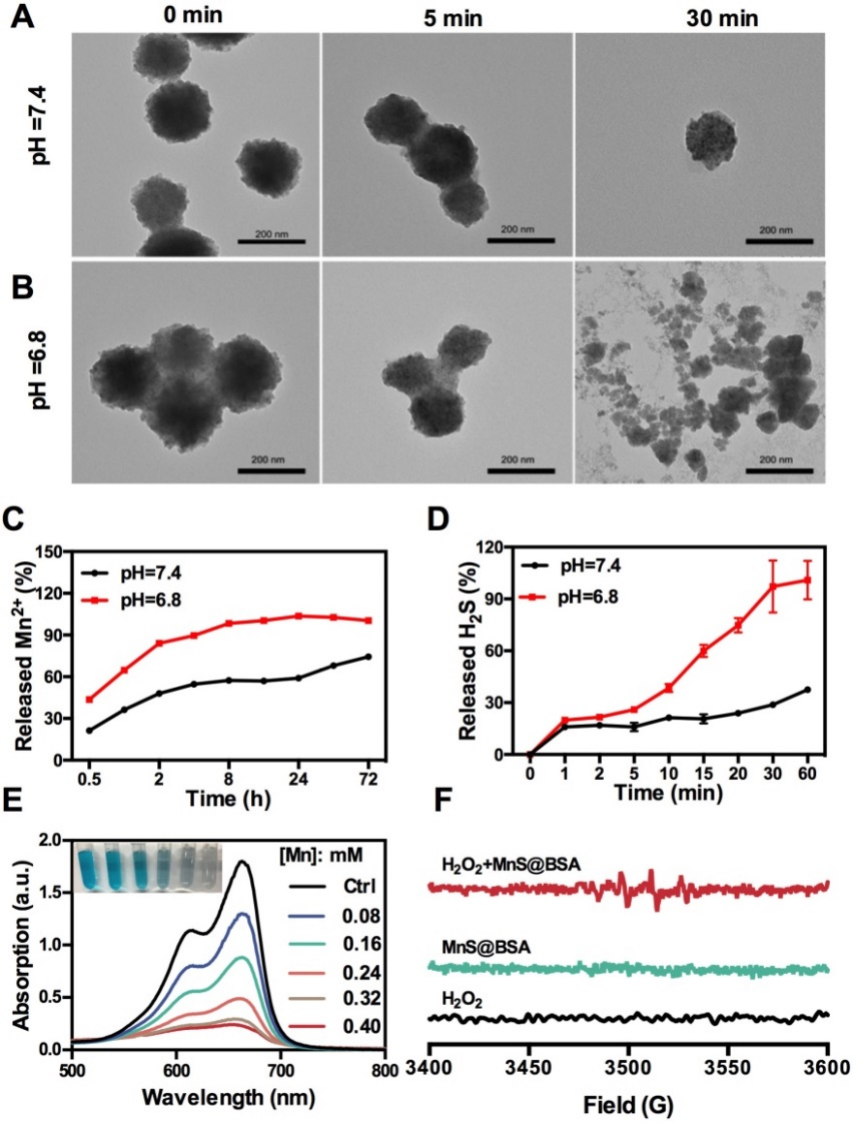
TEM images of MnS@BSA at 0, 5, and 30 min after incubation in HEPES buffer at pH=7.4 (A) and pH= 6.8 (B). (C) Levels of Mn^2+^ released from MnS@BSA in PBS buffer. (D) Levels of H_2_S gas released from MnS@BSA at HEPES buffer of pH=7.4 and pH=6.8 with N_2_ protection. (E) UV-Vis absorption spectra and photographs of methylene blue solutions after different amounts of MnS@BSA adding to H_2_O_2_ (9 mM)/NaHCO_3_ (25 mM) solution for Mn^2+^ catalyzed Fenton-like reaction for 30 min. (F) ESR spectra of H_2_O_2_, MnS@BSA and MnS@BSA + H_2_O_2_ added in NaHCO_3_ (25 mM) solution reacted for 2 h.

The as-synthesized MnS@BSA can be easily decomposed and oxidized in aqueous solutions containing oxygen, especially under acidic conditions. As shown in **Figure 2A** and **2B**, MnS@BSA was degraded much faster in the acidic solution than in the neutral solution. These results are consistent with the levels of Mn^2+^ released from MnS@BSA in PBS buffer at different pH values (**Figure 2C**). After 8 h, 98.3% of MnS@BSA was degraded in acidic solution, while only 57.3% of Mn^2+^ was released at pH 7.4. Afterwards, the degradation of MnS@BSA in 4T1 cells was observed using bio-TEM imaging at 8 and 24 h post-incubation (**Figure S3**). A lot of MnS@BSA were observed inside 4T1 cells after 2 h incubation. After 24 h incubation, sphere MnS@BSA were hardly found in 4T1 cells. These results indicated that MnS@BSA could be degraded in 4T1 cells gradually. Meanwhile, the concentration of released H_2_S from MnS@BSA under different pH conditions was measured using the methylene blue (MB) method. The H_2_S gas from MnS@BSA was released much faster in the acidic solution than in the neutral solution (**Figure 2D**). The concentration of H_2_S can reach up to 12 µM, which is able to kill cancer cells efficiently. These results suggested that pH-responsive MnS@BSA could be fully degraded in aqueous solutions at pH 6.8, followed by the release of Mn^2+^ and H_2_S gas, which promises for cancer diagnosis and treatment.

Manganese element is a trace element in living organisms, which may catalyze Fenton-like reactions in physiological environment to produce highly toxic •OH radicals 36. The Mn^2+^ and HCO_3_^-^ in the blood constitute the catalysts for Fenton reactions, which can consume enriched H_2_O_2_ in tumor microenvironments to generate •OH radials. In order to evaluate the Fenton-like reactivity of MnS@BSA, MnS@BSA was added into MB solution containing HCO_3_^-^ and H_2_O_2_, as shown in **Figure 2E**. The degradation of MB indicated the concentration dependency of manganese concentrations. Both MnCl_2_ (used as a control) and MnS@BSA produced •OH that could degrade MB at a similar rate (**Figure S4**). A unique quadruple peak of •OH was observed in electron spin resonance (ESR) spectra (**Figure 2F**), but was not observed in control groups. All results indicated that released Mn^2+^ ions from MnS@BSA can generate a lot of •OH under physiological conditions.

### *In vitro* Combination Therapy

**Figure 3:**
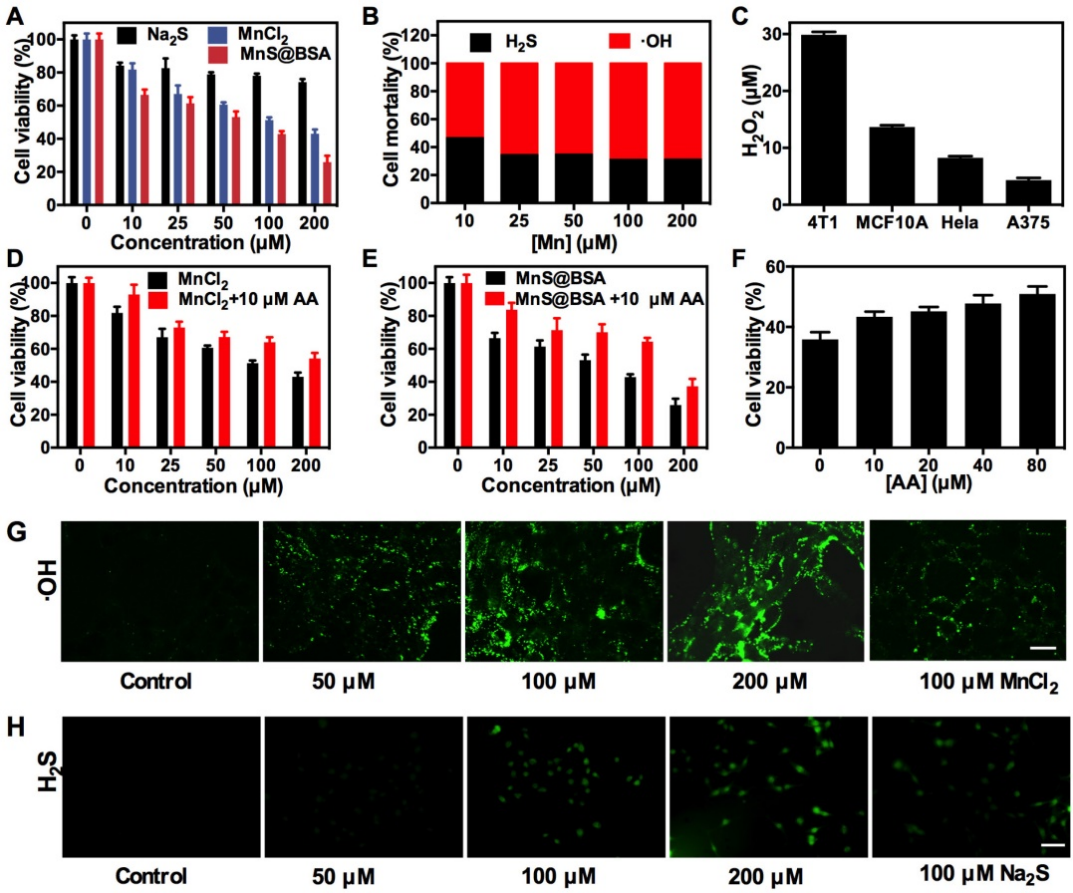
(A) Cell viability of 4T1 cells after incubation with Na_2_S, MnCl_2_ and MnS@BSA at different concentrations for 24 h. (B) Mortality of 4T1 cells caused by •OH radicals and H_2_S gas after incubated with MnS@BSA for 24 h. (C) H_2_O_2_ concentrations in 4T1, MCF10A, HeLa, A375 cells detected by a H_2_O_2_ kit. Viability of 4T1 cells incubated with (D) MnCl_2_, MnCl_2_+10 µM L-ascorbic acid (AA), (E) MnS@BSA, MnS@BSA+10 µM AA. (F) Cytotoxicity of 4T1 cells incubated with 200 µM of MnS@BSA and various concentrations of AA: 0. 10, 20, 40, 80 µM for 24 h. (G) Confocal images of 4T1 cells incubated with various concentration of MnS@BSA: 0, 50, 100, 200 µM and MnCl_2_: 100 µM for 4 h and stained with DCFH-DA fluorescence probe. (H) Fluorescence images of 4T1 cells incubated with WSP-5 H_2_S fluorescence probe for 30 min, subsequently adding various concentration of MnS@BSA :0, 50, 100, 200 µM and Na_2_S 100 µM for 30 min, scale bar 100 µm.

Next, *in vitro* experiments of MnS@BSA were performed to evaluate its combination cancer therapeutic effect and cytotoxicity. As shown in **Figure 3A**, cell viability showed a concentration-dependent therapeutic effect on 4T1 cells when incubated with Na_2_S, MnCl_2_ and MnS@BSA (10-200 µM), which is in agreement with the live/dead cell staining results (**Figure S5**). The therapeutic effect of MnS@BSA on tumor cells can be attributed to the bioeffects of both H_2_S gas and •OH radicals. The mortality of 4T1 cells caused by H_2_S gas and •OH radicals was calculated in **Figure 3B**. As one can see that 65-69% of cytotoxicity was induced by •OH radicals, which suggested •OH radicals were more potent than H_2_S gas at all tested concentrations. Interestingly, Na_2_S, MnCl_2_ and MnS@BSA showed negligible cytotoxicity on MCF10A cells (a control cell line) as evidenced by the maintained cell viability at ~75% at all tested concentrations (10-200 µM) (**Figure S6**). The different cytotoxicity between 4T1 and MCF10A cells was attributed to the levels of H_2_O_2_ inside cells. Indeed, the H_2_O_2_ concentration of 4T1 cells was 2.2 times higher than that of MCF10A (**Figure 3C**), followed by HeLa and A375 cells. This higher concentration of H_2_O_2_ allowed for the enhanced •OH generation for responsive CDT treatment of 4T1 cells but no other cell lines. Therefore, MnS@BSA elicited an improved therapeutic effect on tumor cells with high levels of H_2_O_2_. To further demonstrate the therapeutic mechanism, reductive L-ascorbic acid (AA) was used to neutralize •OH radicals at cell level. By adding 10 µM of AA to different concentrations of MnCl_2_ (**Figure 3D**) or MnS@BSA (**Figure 3E**), 4T1 cell viability could increase by 10-21%. In particular, with the increase of AA, toxic effects of •OH radicals produced by MnS@BSA were gradually decreased (**Figure 3F**). These results indicated that therapeutic effects of MnS@BSA were attributed to the combination effects of •OH radicals and H_2_S gas.

In order to directly observe the generation of •OH radicals *in vitro*, 4T1 cells were incubated with different concentrations of MnS@BSA for 4 h. Then the probe of oxidative stress, 2',7'-dichlorofluorescin diacetate (DCFH-DA), was added. After deacetylates with intracellular esterase to form the non-fluorescent 2',7'-dichlorofluorescin (DCFH), DCFH would be further oxidized into bright green fluorescent dye, 2',7'-dichlorofluorescein (DCF) by reactive oxygen species (ROS) including •OH radicals 36, 49, which can be observed by confocal laser scanning microscopy (CLSM). As expected, the fluorescence intensity of DCF was elevated as the amount of MnS@BSA increased from 50 to 200 µM, which suggested the increase of oxidative stress strongly depended on the concentration of MnS@BSA (**Figure 3G**). These results are consistent with the results of Fenton-like reaction mentioned above. Similarly, free MnCl_2_ also could induce the generation of ROS. Meanwhile, H_2_S gas released in 4T1 cells was observed by adding a Washington State Probe-5 (WSP-5) H_2_S fluorescent probe (**Figure 3H**). It is based on 2-pyridyl disulfide fluorescent, which release the fluorophores and turn on the fluorescence by tandem nucleophilic subsitution-cyclization reaction. WSP-5 probe can selectively and rapidly react with H_2_S in cells to generate a green fluorescence signal 50. The fluorescence intensity of WSP-5 was increased in accordance to the added concentration of MnS@BSA. The same effect was also evidenced in the Na_2_S group, further indicating that MnS@BSA can release H_2_S gas inside cells. All these results proved that MnS@BSA can generate •OH radicals and H_2_S in 4T1 cells, promising for combined CDT and H_2_S gas therapy.

### *In vivo* Imaging and Biodistribution

**Figure 4:**
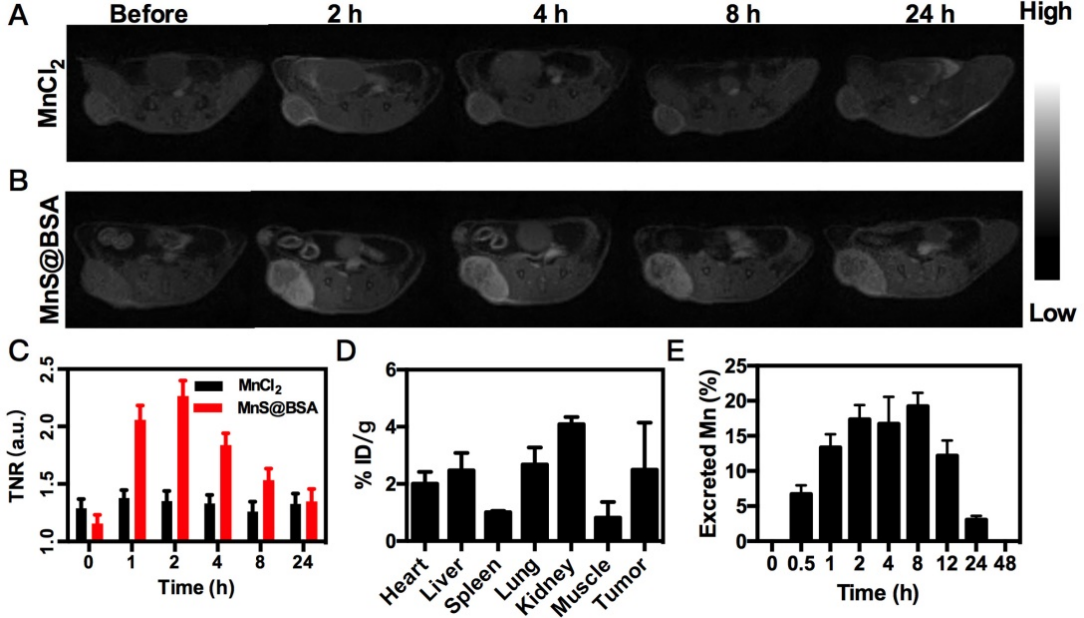
MRI images of 4T1 tumor-bearing mice after i.v. injection of MnCl_2_ (A), MnS@BSA (B) at various time points. (C) The TNR contrast ratio after i.v. injection of MnS@BSA or MnCl_2_. (D) Biodistribution of manganese element after injection of MnS@BSA at 24 h post-injection. (E) The content of manganese element in mice excrements after injection of MnS@BSA over time.

The released Mn^2+^ ions from MnS@BSA are not only a catalyst for the Fenton-like reaction, but also a contrast agent for tumor pH-responsive* T*_1_-weighted MRI. Since MnS@BSA is pH-responsive in acidic solutions, its longitudinal relaxivity (r_1_) was measured under different pH conditions, as shown in **Figure S7**. The *T*_1_ relaxivity of MnS@BSA, r_1_, was calculated to be 23.67 mM^-1^s^-1^ in a pH 6.8 buffer at 2 h. Encouraged by the high r_1_ value of MnS@BSA at pH 6.8, we performed *in vivo* tumor pH-responsive MRI of MnS@BSA on 4T1 tumor-bearing mice after intravenous (i.v.) injection with MnCl_2_ as a control. Tumor signal of 4T1 bearing mice treated with MnS@BSA clearly increased over time, while the tumor signal kept no change for mice treated with MnCl_2_ (**Figure 4A** and** B**). The tumor to normal ratio (TNR) of mice treated with MnS@BSA reached to a peak number of 2.26 at 2 h post-injection (**Figure 4C**). The mice treated with MnCl_2_ showed negligible MR contrast enhancement. These results were further confirmed by the biodistribution study of Mn element at 4 h post-injection (**Figure 4D**). Since Mn^2+^ can be gradually excreted through metabolism, we also monitored Mn levels in excrements (**Figure 4E**). At 48 h post-injection, Mn concentration in mice was reduced to 0.02%, suggesting that MnS@BSA could be fully excreted within one day. These results suggested MnS@BSA could be fully cleared from the body of mice and exempt from the risk of potential long-term toxicity.

### *In vivo* Combination Therapy

**Figure 5:**
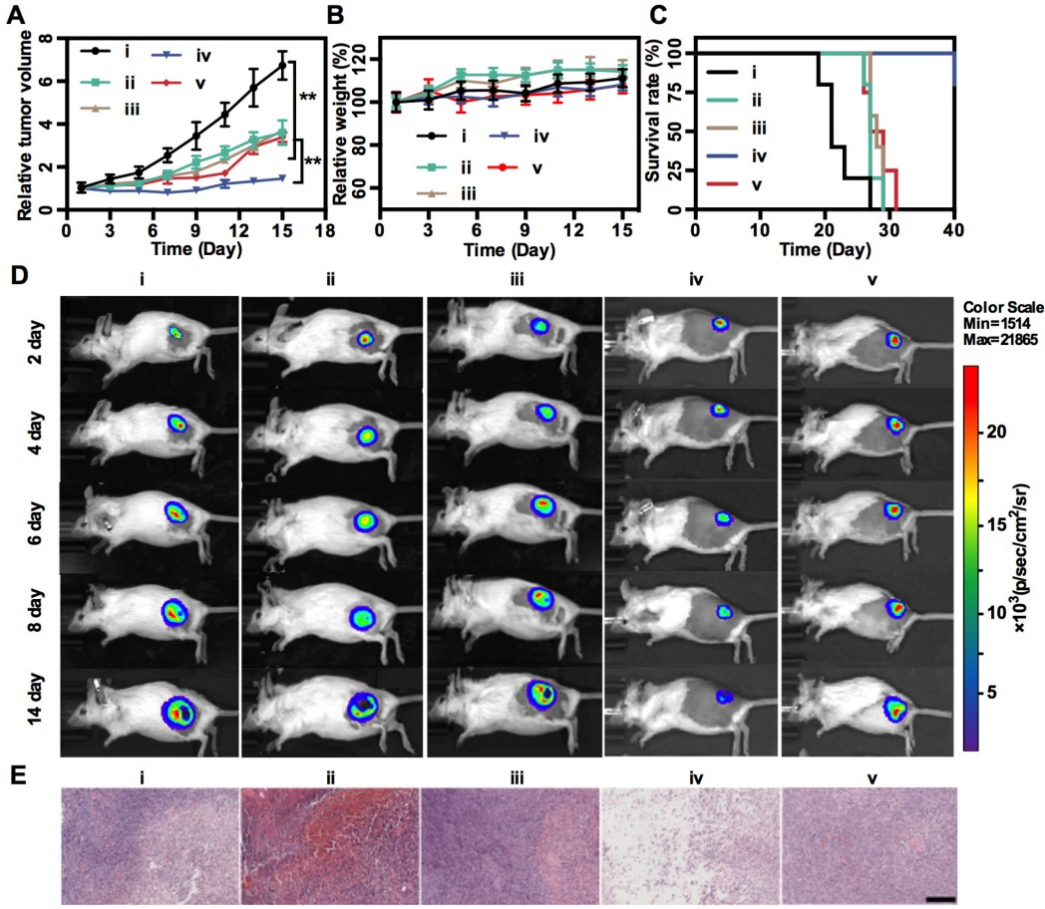
*In vivo* treatment. Tumor growth profiles (A), mice weight changes (B), and survival rates (C) of 4T1-luc tumor-bearing mice in different treatment groups: (i) Saline, (ii) MnCl_2_, (iii) Na_2_S, (iv) MnS@BSA, (v) MnS@BSA+AA. (D) *In vivo* bioluminescence images of 4T1-luc tumor-bearing mice in 5 groups throughout the treatment. (E) H&E staining images of tumors in all experiments groups. Scale bar: 200 µm, **p<0.05.

Finally, *in vivo* MnS@BSA-induced therapeutic effect was studied on 4T1-luc tumor-bearing mice. Mice were randomly divided into 5 groups: (i) saline, (ii) MnCl_2_, (iii) Na_2_S, (iv) MnS@BSA, (v) MnS@BSA+AA. The tumor volumes were monitored every 2 d during 2 weeks and normalized to their initial size in **Figure 5A**. The saline control group showed fast tumor growth, whereas the MnS@BSA treated group exhibited higher tumor suppression as compared with MnCl_2_ and Na_2_S treated group. The MnS@BSA+AA treated group exhibited obvious tumor regrowth after day 11, due to the neutralization of •OH radicals by AA. These results suggested the combination effect of CDT/gas therapy over any single modality treatment solely. For all groups, mice showed no noticeable body weight change, suggesting no systemic toxicity of MnS@BSA (**Figure 5B**). Meanwhile, the survival of mice administered with MnS@BSA was greatly prolonged (**Figure 5C**). More importantly, no obvious toxicity was induced by MnS@BSA, as evidenced by results of blood biochemistry, hematology analysis, and acute toxicity measurement of all major organs (**Figure S8-10**). Furthermore, all tumor growth was also monitored using bioluminescence imaging during treatment period. As shown in **Figure 5D,** the tumor luminescence was located on right hind of all groups in the whole treatment process. Especially, only the tumor of MnS@BSA group showed obviously suppression effect, but tumors of other groups kept growth. After different treatments, H&E staining images of all treatment groups were taken (**Figure 5E**). Tumors of saline, MnCl_2_ and Na_2_S groups show more chromatin and large nuclei, indicated silght effect of tumor cell proliferation. But for MnS@BSA treated group, the tumor slices shown that the chromatin was pyknotic or even absent in the slice. MnS@BSA-AA group can consume some ·OH to reduce the oxidative damage, which show more chromatin than MnS@BSA group. The tumors treated with CDT/gas combination therapy showed much higher damage than other groups.

## Conclusions

In conclusion, we have developed a tumor pH-responsive metastable-phase MnS@BSA for combined CDT and H_2_S gas therapy. The as-prepared metastable-phase MnS@BSA is able to response mildly acidic microenvironment and can release Mn^2+^ for Fenton-like reaction to generate •OH at the presence of endogenous H_2_O_2_ of tumor cells. The MnS@BSA can also generate H_2_S to benefit gas therapy of cancer. Furthermore, the size-controllable MnS NPs can be used as an MRI contrast agent for treatment monitoring owing to its high r_1_ relaxivity of Mn^2+^ ions. This work provides a new design strategy of a nanotheranostic agent for traceable H_2_S gas therapy ydrogen primed CDT. Our findings open new horizons for the biomedical applications of manganese-based cancer theranostics.
