# Supplementary material for: Tumor pH-responsive metastable-phase manganese sulfide nanotheranostics for traceable hydrogen sulfide gas therapy primed chemodynamic therapy
Source: Theranostics. 2020 Jan 20;10(6):2453–62. doi: 10.7150/thno.42981 (PMC7052883; doi:10.7150/thno.42981)
Supplement: Supplementary file 1 — Supplementary figures and tables. [file thnov10p2453s1.pdf]

# Supporting Information

## Tumor pH-responsive metastable-phase manganese sulfide nanotheranostics for traceable hydrogen sulfide gas therapy primed chemodynamic therapy

Ting He[a,b‡], Xialing Qin[a‡], Chao Jiang[a], Dawei Jiang[a], Shan Lei[a], Jing Lin[a], Wei-Guo Zhu[c], Junle Qu[b], Peng Huang[a]*

[a] Marshall Laboratory of Biomedical Engineering, International Cancer Center, Laboratory of Evolutionary Theranostics (LET), School of Biomedical Engineering, Shenzhen University Health Science Center, Shenzhen 518060, China

[b] Key Laboratory of Optoelectronic Devices and Systems of Ministry of Education and Guangdong Province, College of Optoelectronic Engineering, Shenzhen University, Shenzhen 518060, China

[c]Guangdong Key Laboratory of Genome Instability and Human Disease Prevention, Carson International Cancer Center, Department of Biochemistry and Molecular Biology, Shenzhen University School of Medicine, Shenzhen 518055, China.

**Corresponding author:** Peng Huang

**Addresses:** 1066 Xueyuan Boulevard Shenzhen University Health Science Center, Shenzhen, 518060, China. E-mail: peng.huang@szu.edu.cn (Peng Huang)

‡ These authors contributed equally to this work.

**Experimental Details**

**Materials.** All regents and chemicals were commercial available. $Mn(NO_3)_2 \cdot 4H_2O$ (98%), Bovine serum albumin (BSA, 98%), $NaHCO_3$ (99.5%), Dimethyl Pyridine N-oxide (97%), Thiazole Blue (97.5%), N, N-Dimethyl-p-phenylenediamine sulfate(DMPD) (99.8%) were purchased from Sigma-Aldrich (St. Louis, MO, USA). $H_2O_2$ (30 wt%) was purchased from Aladdin Reagent (Shanghai, China). Dimethyl sulfoxide (99.8%) and Methyl Blue (MB, 95%) were purchased from J&K Chemical Ltd. (Shanghai, China). $FeCl_3 \cdot 6H_2O$ (99%) were purchased from Macklin Biochemical Co., Ltd (Shanghai, China). $H_2SO_4$ ( 95%-98%), HCl (95%-98%) and $Zn(OAc)_2 \cdot 2H_2O$(99%) were purchased from Chengdu Jinshan Chemical Co., Ltd. (Chendu, China) $Na_2S \cdot 9H_2O$ (98.0%) was purchased from Xilong Scientific Co., Ltd.(Guangdong China), Hydrogen Peroxide Assay Kit (S0038) was purchased from Beyotime Biotechnology (Shanghai, China), Reactive oxygen species (ROS) detection probe 2',7'-dichlorohydrofluorescin diacetate ($H_2$DCFDA) (97%) was purchased from Dalian Meilun Biotechnology Co., Ltd.(Liaoning, China), $H_2S$ Fluorescent Probe WPS-5 was purchased from Shanghai Maokang Biotechnology Co., Ltd. (Shanghai, China), D-Luciferin Potassium Salt was purchased from PerkinElmer (MA, USA).

Deionized water (18.2 MΩ·cm) was used in all experiments, which was produced by a Milli-Q Academic water purification system (Millipore Corp., Billerica, MA, USA). Reagents for cell culture were supplied by Gibco (Tulsa, OK, USA), DMEM medium, Fetal calf serum, penicillin (100 U $mL^{-1}$) and streptomycin (100 g $mL^{-1}$), incubation at 37 °C with 5% $CO_2$.

**Instruments.**

UV-Vis-NIR spectra were recorded on a Varian UV-Vis-NIR spectrophotometer (Cary 60 Bio, USA). Cell viability assessment was recorded by a synergy H1 microplate reader (BioTEK,

USA). Transmission electron microscopy (TEM) images were obtained using a HT7700 instrument (Hitachi Electronics, Japan). Scanning electron microscopy (SEM) imaging and energy-dispersive spectrometry (EDS) were analyzed using a ZEISS SUPRA® 55 instrument (Carl Zeiss, Germany). The pH values were measured by a digital pH-meter (ThermoFisher Scientific, USA).

X-ray diffractometer (XRD)-D8 (Bruker, Germany)was used to detect the crystal structure of MnS@BSA. Mn concentration was quantified by an Inductively Coupled Plasma Atomic Emission Spectrometry (ICP-AES) JY2000-2 (Horiba Jobin Yvon, France). X-ray Photoelectron spectroscopy (XPS) was recorded on Thermo Scientific X-ray Photoelectron Spectroscopy (K-Alpha+). Cellular fluorescence of ROS was measured by a Laser Confocal Microscope TCS SP5II machine (Leica, German). Cellular fluorescence of $H_2S$ was obtained by an inverted fluorescent microscope NIKON TS2 (Nikon, Japan).

*In vivo* MRI was performed using a magnetic resonance imager UMR770 (United-Imaging, China). Bioluminescence imaging was accomplished using an IVIS® Spectrum system (Perkinelmer, USA) (Ex/Em 460/520 nm)

***In vitro* degradation of MnS@BSA** 50 μL of MnS@BSA (5.5 mM) was dissolved in 500 μL of HEPES buffer (10 mM, pH 6.8 or pH 7.4), and their morphology was observed at 5, 30 and 60 min after incubation using bio-TEM.

**$T_1$ relaxivity of MnS@BSA** MnS@BSA with different Mn element concentration (0.05, 0.1, 0.2, 0.4 mM) were dispersed in HEPES solutions with different pH values (6.8, 7.4) for 2 h. Then the $T_1$ relaxivity was detected by 3 T clinical MRI scanner (UMR 790, United-Imaging, Shanghai, China). An fast spin echo (FSE) sequence with 19 different values of $T_1$ (TR (repetition time)=5000 ms; TE (ehco time) =100, 150, 200, 250, 300, 350, 400, 450, 500, 550,

600, 700, 800, 900, 1000, 1200, 1300, 1500, 2000 ms) for $T_1$ measurements. $T_1$ relaxation times were calculated by fitting the signal intensities with increasing $T_1$ to $I_{(t)}=I_0[1-k\bullet\exp(-t/T_1)]$ by using a nonlinear least-squares fit of the Levenberg-Marquardt algorithm.

**Hemolysis of MnS@BSA** Red blood cells (RBCs) were isolated from mouse serum by centrifugating a mixture containing 0.5 mL of the blood sample and 1 mL of PBS solution at 4500 rpm for 3-5 min. Then an aloquat of the diluted RBC suspension (0.3 mL) was added to quadruple volume of PBS solution with different concentrations of MnS@BSA ([Mn]=10, 25, 50, 100, and 200 μM). The mixture was vortexed and left for precipitation for 3 h at room temperature. Samples were then centrifuged to measure the OD of the supernatant at 541 nm by UV-Vis-NIR spectrophotometer. RBCs treated with deionized water were used as the negative group and PBS as the positive control.

**Calcein-AM/PI staining**

4T1 cells were treated with $Na_2S$, $MnCl_2$ and MnS@BSA at the same Mn concentration at 200 μM for 48 h. Then the cells were incubated with 4 μM of Calcein-AM/PI for 30 min. Subsequently, the fluorescence images were acquired by an Olympus IX81 inverted fluorescence microscope (Olympus, Tokyo, Japan).

**Animals** BALB mice (31-45 days, 18-22 g), were purchased from the Animal Experimental Center of Guangdong Province and were housed at the Experimental Animal Center of Shenzhen Research Institute of Peking University. All animal experiments were approved by the Animal Ethics Committee of Shenzhen University and complied with relevant policies and regulations.

***In vivo* pharmacokinetics of MnS@BSA** For *in vivo* evaluation of pharmacokinetic profiles, three healthy mice were administered with 2.5 mg/kg of MnS@BSA via tail vein injection, and blood was collected at 0, 0.5, 1, 2, 4, 8, 12, and 24 h post-injection. 0.5 mL of nitric acid was

added into each blood sample for overnight incubation, and the final volume was adjusted to 1.5 mL. The concentration of Mn was then measured by ICP-AES.

***In vivo* toxicity of MnS@BSA** For *in vivo* biosafety, five healthy mice were administered with 2.5 mg/kg of MnS@BSA through the tail vein, and the blood was collected (about 200 μL) at day 0, 7 and 15 post-injections. The blood was sat at room temperature for 4 h and centrifuged at 5000 rpm for 10 min, the supernatant was stored at -20 °C. The following parameters were tested: alanine aminotransferase (ALT), aspartate aminotransferase (AST), blood urea nitrogen (BUN) and creatinine (Cr).

**Acute toxicity evaluation** Three healthy mice were administered with 2.5 mg/kg of MnS@BSA through the tail vein and then euthanized at 2 h post-injection. The heart, liver, spleen, lung, and kidneys were collected and fixed in paraformaldehyde for HE staining to perform histological analysis.

**Statistical analysis** The statistical significance of differences between control and experimental groups were determined by two-sided student *t* test. All statistical tests were two-sided and P values less than 0.5 were considered statistically significant. All statistical analyses were performed using GraphPad Prism of 6.0 software.

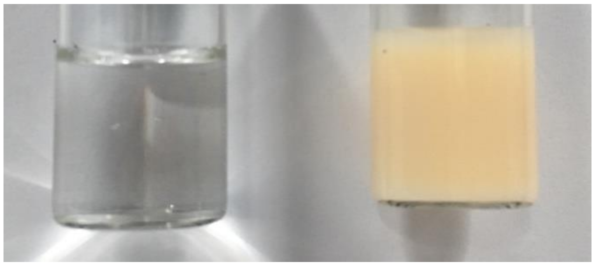

**Figure S1** Photograph of before and after synthesized MnS@BSA.

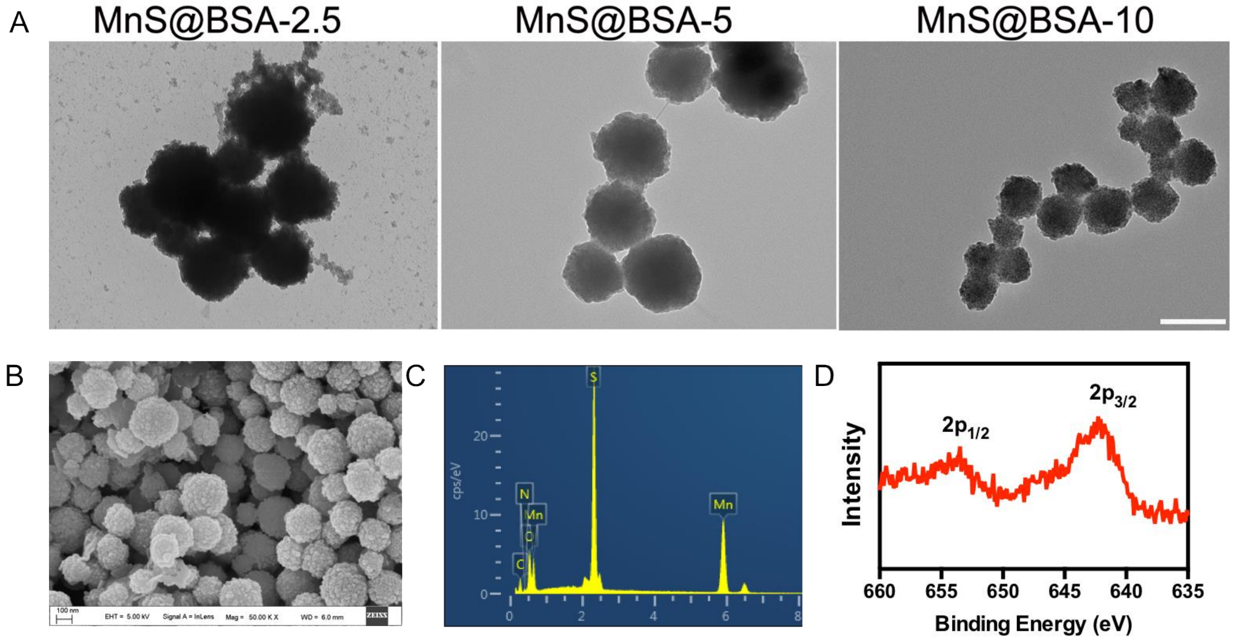


**Figure S2** (A) TEM images of MnS@BSA-2.5, MnS@BSA-5, MnS@BSA-10 (Numbers represent the amount of BSA added). (B) A representative SEM image of MnS@BSA-10. (C) EDS and (D)XPS spectrum of Mn 2p for MnS@BSA-10. Scale bar 200 nm.

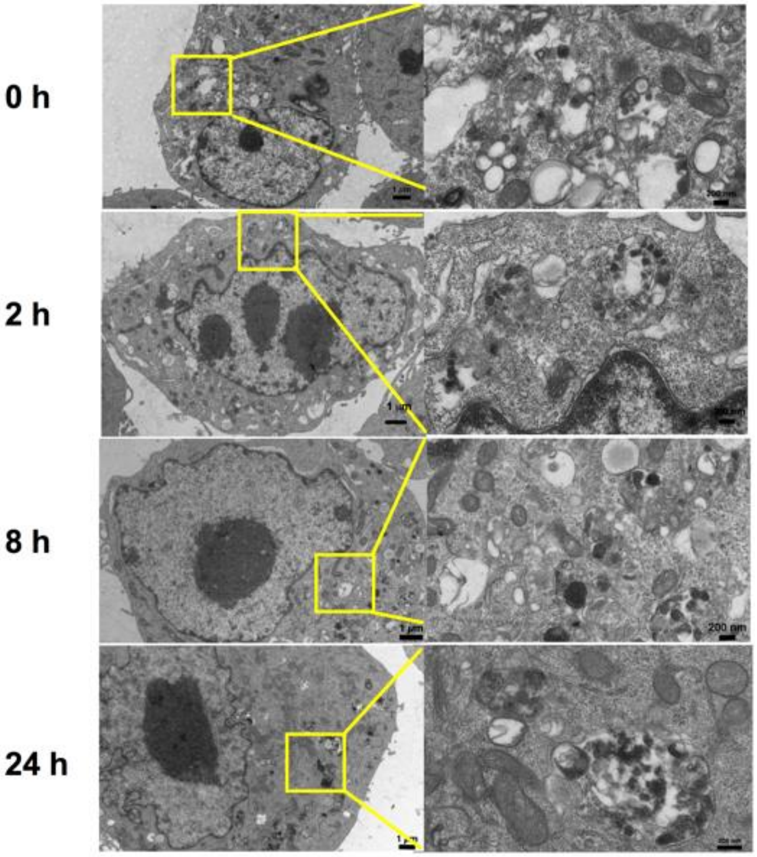


**Figure S3** Representative bio-TEM images of 4T1 cells incubated with MnS@BSA (200 μM) for 0, 2, 8, and 24 h. Scale bars, 1 μm and 200 nm, respectively.

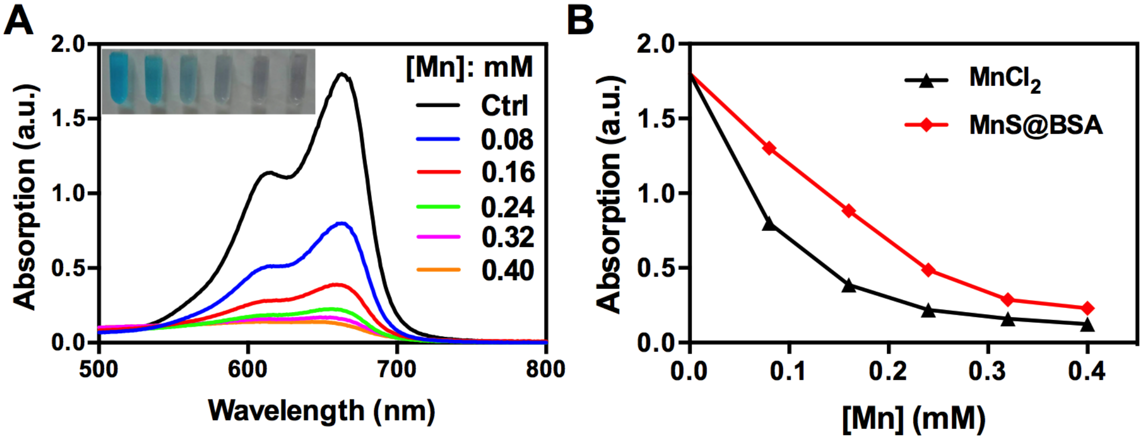

**Figure S4** (A) UV-Vis absorption spectra and digital photos of methylene blue solutions after adding different concentrations of $Mn^{2+}$ into $H_2O_2$ (9 mM) / $NaHCO_3$ (25 mM) solutions for 30 min. (B) UV-Vis absorbance of methylene blue solution after 30 min.

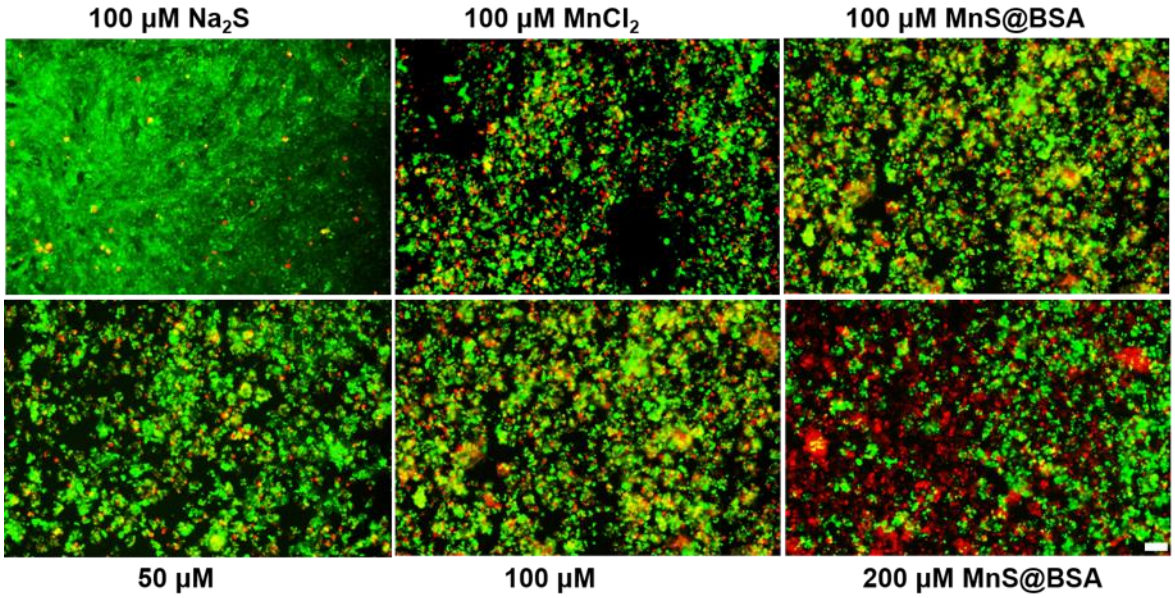


**Figure S5** Live/dead staining of 4T1 cells incubated with $Na_2S$, $MnCl_2$, or MnS@BSA, scalebar:200 μm.

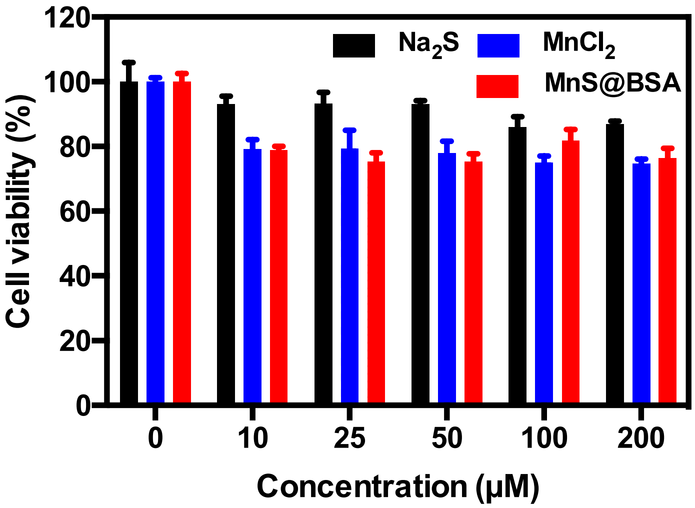


**Figure S6** Cell viability of MCF10A cells incubated with $Na_2S$, $MnCl_2$, or MnS@BSA for 24 h.

| pH=7.4 | pH=6.8 |
|---|---|
| Y = 14.18*X + 0.7246 | Y = 23.67*X + 3.167 |

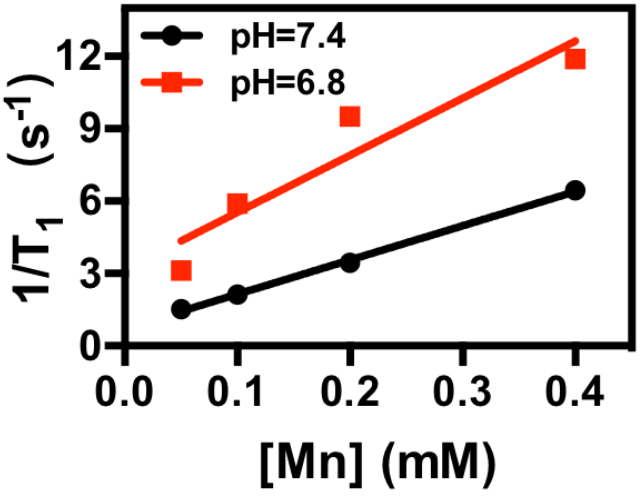


**Figure S7** $T_1$ relaxivity of MnS@BSA in HEPES solutions at different pH values (pH=6.8 and pH=7.4).

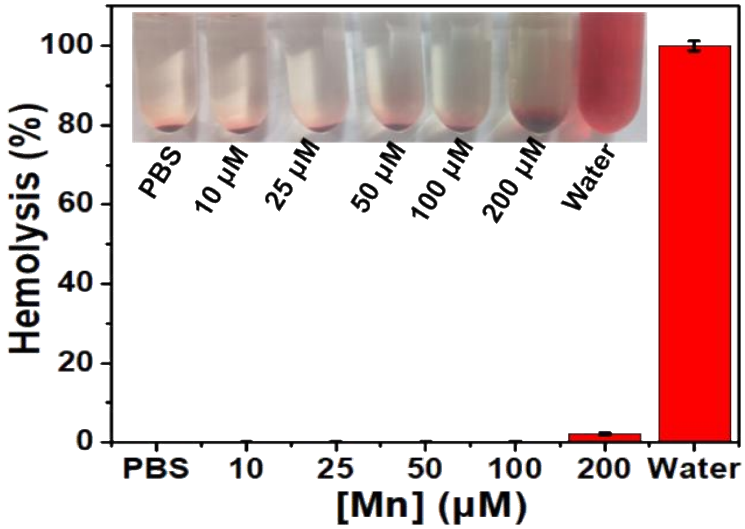


**Figure S8** Hemolysis assay of MnS@BSA at different concentrations.

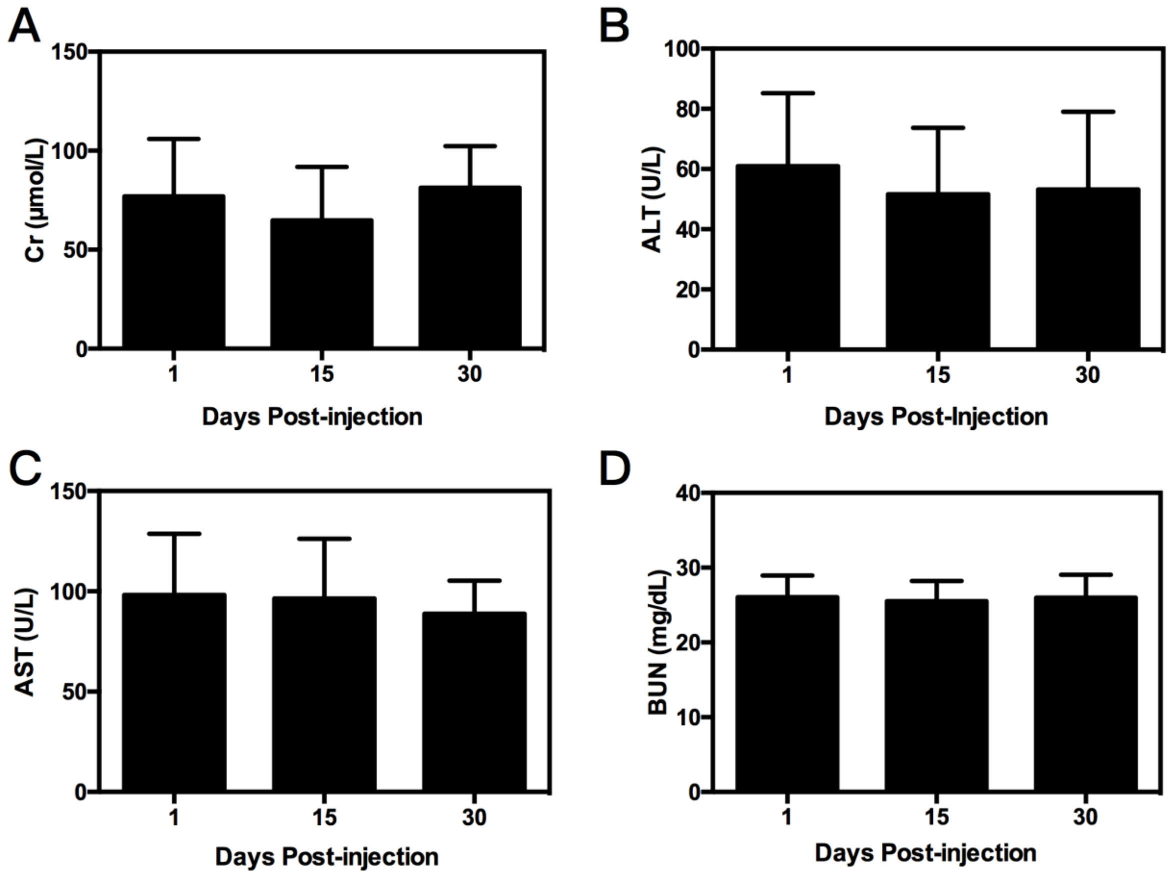


**Figure S9** Systemic toxicity evaluation of MnS@BSA: (A) Cr, (B) ALT, (C) AST, (D) BUN.

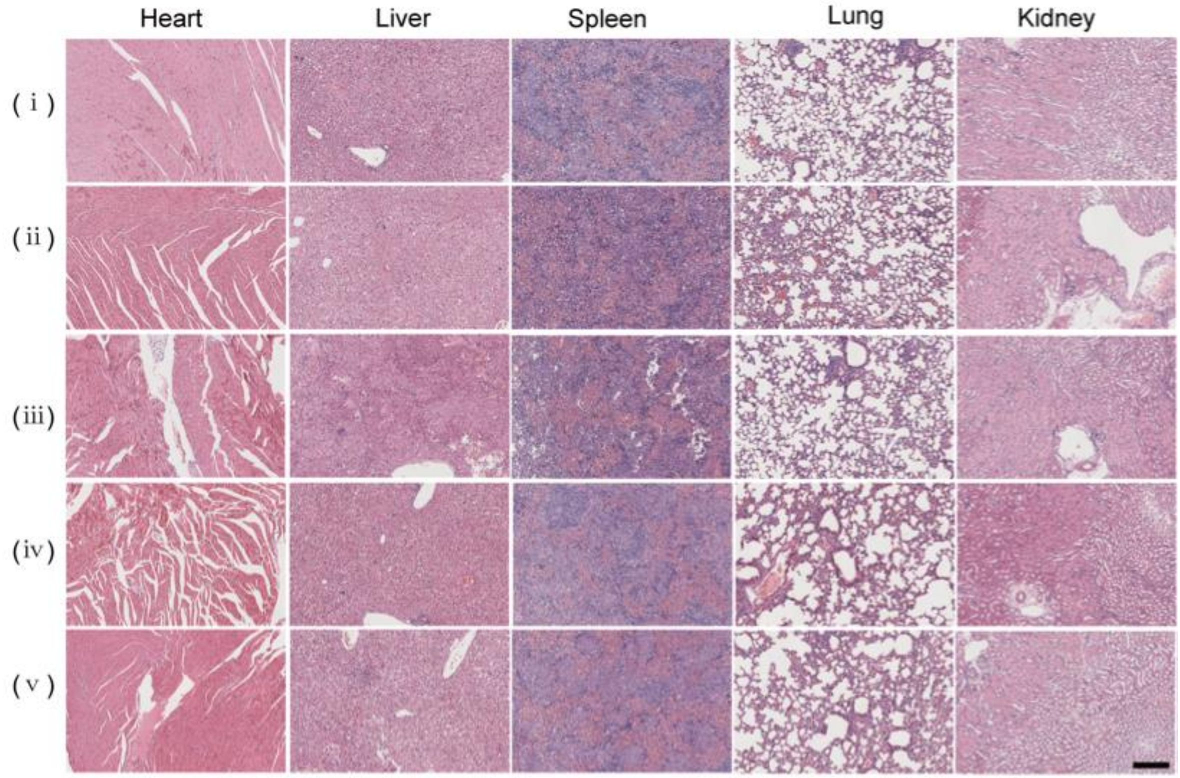


**Figure S10** Hematoxylin and eosin (HE) staining of main organs from all experiments groups: (i) saline, (ii) $MnCl_2$, (iii) $Na_2S$, (iv) MnS@BSA, (v) MnS@BSA+AA. Scale bar: 200 µm.
